# Impact of a First-Order Riparian Zone on Nitrogen Removal and Export from an Agricultural Ecosystem

**DOI:** 10.1100/tsw.2001.380

**Published:** 2001-11-21

**Authors:** J.T. Angier, G.W. McCarty, T.J. Gish, C.S.T. Daughtry

**Affiliations:** USDA-ARS Environmental Quality Lab, Beltsville, MD 20705, USA

**Keywords:** denitrification, groundwater, preferential flow, wetland

## Abstract

Riparian zones are reputed to be effective at preventing export of agricultural groundwater nitrogen (N) from local ecosystems. This is one impetus behind riparian zone regulations and initiatives. However, riparian zone function can vary under different conditions, with varying impacts on the regional (and ultimately global) environment. Rates of groundwater delivery to the surface appear to have significant effects on the N-removing capabilities of a riparian zone. Research conducted at a first-order agricultural watershed with a well-defined riparian zone in the Maryland coastal plain indicates that more than 2.5 kg/day of nitrate-N can be exported under moderate-to-high stream baseflow conditions. The total nitrate-N load that exits the system increases with increasing flow not simply because of the greater volume of water export. Stream water nitrate-N concentrations also increase by more than an order of magnitude as flow increases, at least during baseflow. This appears to be largely the result of changes in dominant groundwater delivery mechanisms. Higher rates of groundwater exfiltration lessen the contact time between nitrate-carrying groundwater and potentially reducing riparian soils. Subsurface preferential flow paths, in the wetland and adjacent field, also strongly influence N removal. Simple assumptions regarding riparian zone function may be inadequate because of complexities observed in response to changing hydrologic conditions.

### INTRODUCTION

Riparian buffer zones (vegetated areas around streams) may be effective natural remediation sites for agricultural contaminants. Putative riparian zone remediative functions include retention of phosphorus (P) from surface runoff water (with the riparian zone acting as a net sink for eroded sediments) and removal of nitrogen (N) from groundwater. Each of these functions requires certain conditions for optimal efficacy. Sediment trapping and P retention behavior in riparian zones are measurable in the field, at least in the short term. Groundwater processes, which strongly affect N transport and fate, are more difficult to discern. Various riparian zone regulations and initiatives are formulated using an incomplete knowledge of riparian zone processes and function, and thus may be inadequate for long-term environmental protection.

Small, first-order streams (streams with no tributaries) and their associated riparian corridors have received particular attention as sites for effective removal of N from the ecosystem[1,2]. These environments are thought to play a role in N transformations greatly disproportionate to their small percentage of the total landscape[2,3]. This study was conducted partly to examine the effectiveness of a first-order riparian zone at N removal. Complex interactions between hydrology and biogeochemistry appear to affect the overall function of the riparian system, with a resulting high degree of variability in N transformation and movement observed.

### Nitrogen

Nitrate-N export from agricultural areas occurs primarily through groundwater. N applied to fields migrates through the vadose zone and enters the water table, where its mobility and persistence are primarily determined by groundwater movement and biogeochemical conditions. If conditions within the saturated zone remain oxic, N can remain in the groundwater for long periods[4,5,6]. Eventually, N-carrying groundwater can be discharged into surface waters. If the groundwater passes through an anoxic zone with suitable electron donors (usually C, $Fe^{2+}$, or S) and sufficient microbial populations, there is the potential for nitrate removal[6,7,8]. Riparian buffer strips are environments that should provide these conditions. In addition, dense vegetation commonly found in these areas provides another mechanism, plant uptake, for the removal of N from groundwater[9,10]. However, hydrologic conditions within riparian environments may ultimately determine the extent to which N removal can occur[10,11]. Even under conditions favorable to nitrate removal, such as the presence of fine-grained carbon-rich soils with reducing conditions near the surface (common in riparian environments), preferential subsurface flow can substantially diminish the N-removing capabilities of the system. The greater the deviation from uniform piston-like groundwater flow through the soil matrix, the lower the N mitigation potential of the system is likely to be. Macropores, which are usually abundant (due to extensive rooting systems and prodigious animal burrowing) in riparian wetlands, provide preferential conduits for groundwater flow. Subsurface heterogeneities, particularly zones of higher-than-average hydraulic conductivity, can also serve as preferential flow pathways.

### STUDY SITE

The study site, part of the USDA-Beltsville Agricultural Research Center in Maryland, lies within the mid-Atlantic coastal plain. It is a small agricultural watershed (including about 35 ha of crop land) containing a first-order stream and associated riparian corridor. The first-order stream length is about 1200 m; the forested riparian buffer varies from a minimum width of 60 m to more than 250 m. Fig. 1 is a three-dimensional surface topography map of the study watershed, showing the location of many key features. Fig. 2 is a plan-view topographic map of the entire study site. It shows the orientation of the stream, the locations of the permanent stream sampling/monitoring stations, forest boundary, piezometers, secondary channels, and zones of groundwater exfiltration. Predicted preferential groundwater flowpaths are shown for the upper portion of the agricultural field. The riparian floodplain sediments consist of a 2-m-deep histosol overlying an oxic sand aquifer. The geomorphological characteristics of this site are comparable to other first-order streams in this part of the mid-Atlantic coastal plain, and the site lies at an intermediate overall elevation (30 to 40 m above sea level) compared with other first-order basins in this region.

### METHODS

#### Surface Water Measurements

Sampling stations were constructed at five points along the stream. This allowed separate stream lengths to be monitored individu-

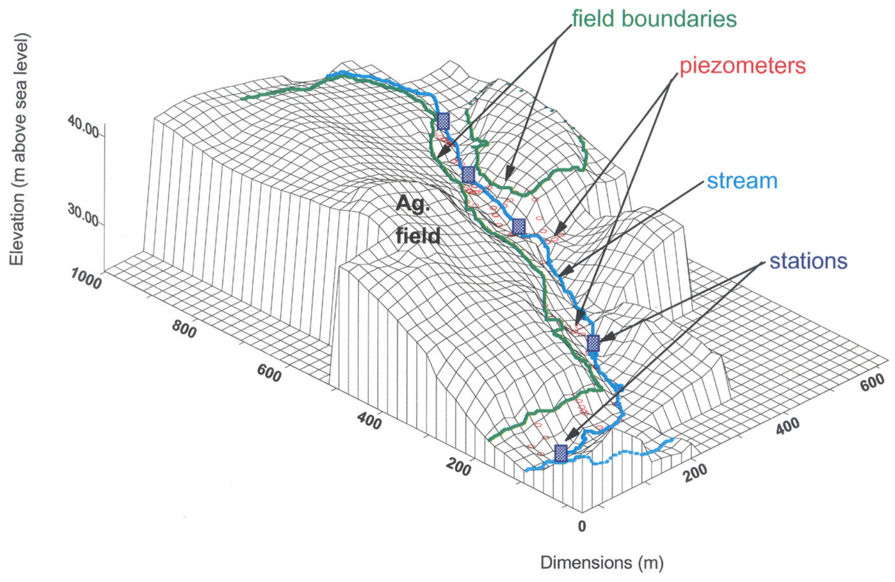

**FIGURE 1.** Three-dimensional view of watershed surface topography. This figure shows location of field edge, stream, sampling stations, and piezometers. The entire first-order stream length is about 1200 m. Riparian zone lies between fields, alongside stream. Coordinates are in meters (origin at SW corner of catchment).

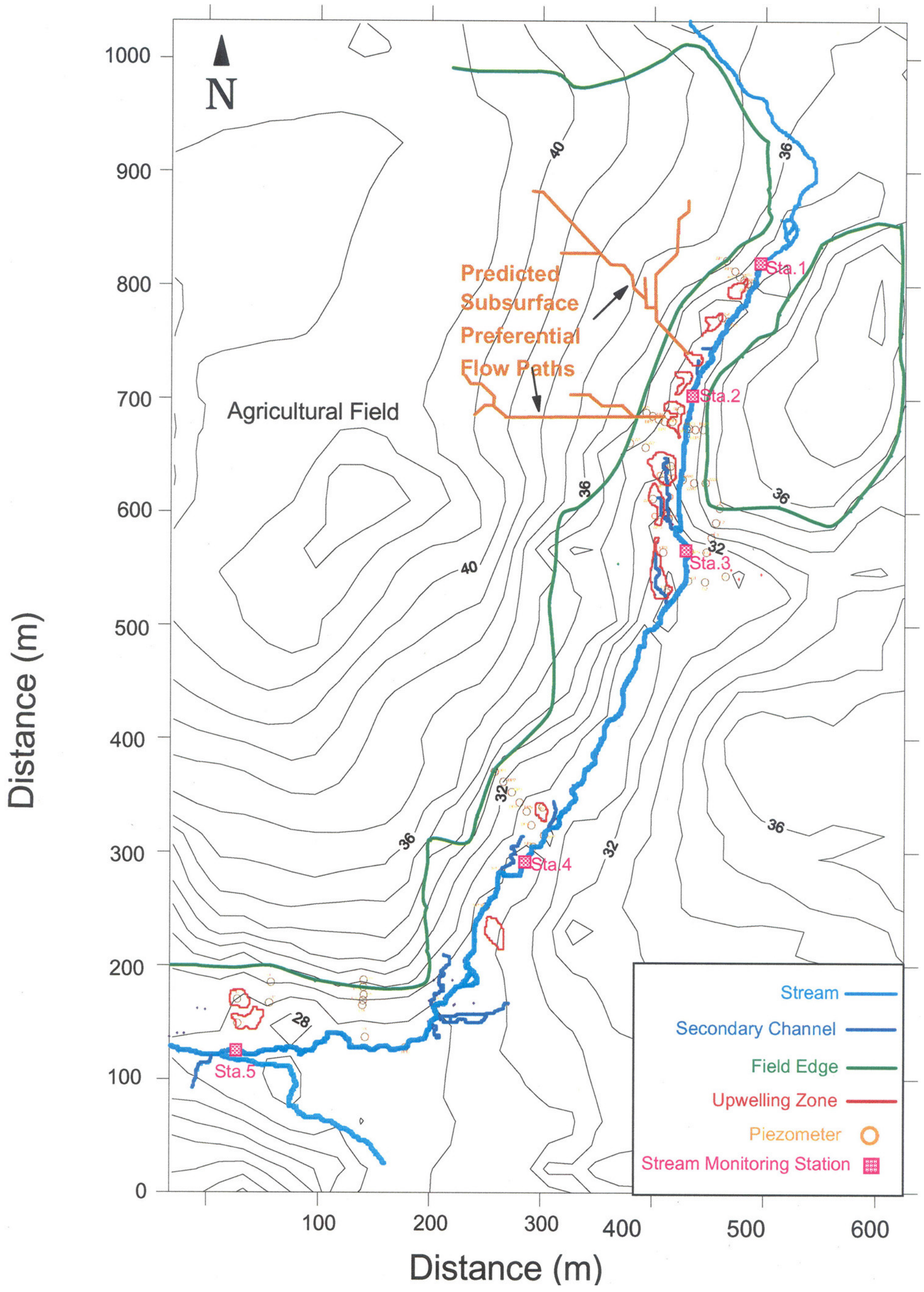

**FIGURE 2.** Plan-view topographic map of entire study site. This figure shows orientation of stream, locations of sampling stations, forest boundary, upwelling zones, piezometers, and secondary channels. Note the preponderance of secondary channels and upwelling zones in the upper portion of riparian corridor. Possible preferential flowpaths beneath field are shown for upper catchment (see Walthall et al.[12]). Coordinates are in meters (origin at SW corner of catchment). Compiled from survey, GPS, and GPR data.

ally and compared. The five permanent measuring and sampling stations installed within the stream channel consist of sheet metal overlying a wooden skeleton. V-notch weirs were added to the front of each channel structure. For stations 1 and 2, where stream flow is lowest, a 60° V-notch weir was used. Stations 3 and 4 are equipped with a 90° V-notch, and at station 5 a 120° V-notch was used. A Sigma 500 Autosampler was used for recording stream levels. A water level/stream discharge relationship (rating curve) was established for each station. This was done by taking frequent measurements with a bucket and stopwatch to accurately determine the discharge (Q) for each stream water level. Discharges in the secondary channels were evaluated the same way, with a small, portable weir temporarily placed in the subchannel. The time required for a given volume of water to flow from the upper part of the stream to the watershed discharge varied, but averaged about 8 h. Evaporation from the stream channel should be negligible given the short residence time and fully covering tree canopy.

### Groundwater Measurements

The field site is instrumented with more than 170 piezometers (2.5-cm PVC pipe with 20-cm screened intervals), mostly in nested transects. Nests (clusters of wells with screened intervals at depths varying from 50 to 350 cm) consist of anywhere from two to seven piezometers each. Transects were instrumented with piezometers from the field edges to the stream between stream stations. Nested piezometers were also placed within the stream channel at regular intervals. Shallow piezometers (within the soil zone) were added when it became apparent that the hydrology of the soil zone differed greatly from place to place. The upper portion of the riparian zone has been instrumented especially thoroughly because of the high degree of spatial heterogeneity observed in this area. Piezometers were sampled approximately every 3 months, with more frequent sampling in selected piezometers within the upper part of the riparian zone.

### Chemical Analysis

Samples were collected along with stream discharge measurements, so contaminant fluxes from each point could also be calculated. Samples were refrigerated until analysis, and carefully decanted into vials after settling. No filtration was performed. Anion ($NO_3^-$, $SO_4^{2-}$, $Cl^-$) analysis of stream and groundwater was done using a Dionex ion chromatograph (IC).

## RESULTS AND DISCUSSION

### Spatial and Temporal Changes in Stream Discharge

Stream flow at this site varies greatly both temporally and spatially, and stream flow characteristics are very different in the upper (stations 1, 2, and 3) and lower (stations 4 and 5) sections of the catchment. Fig. 3 shows stream flows at stations 2, 3, 4,

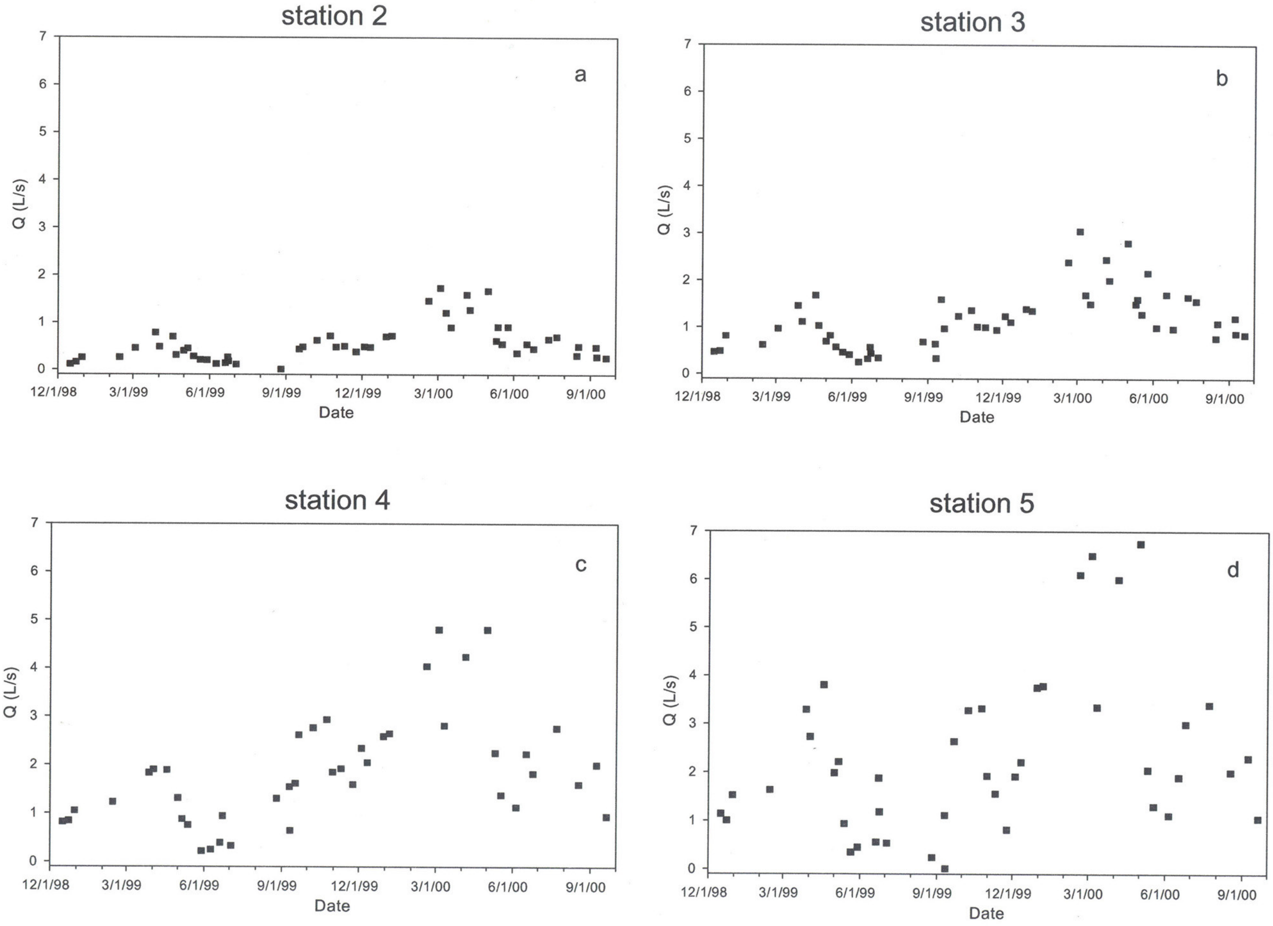

**FIGURE 3.** Stream discharge: station 2 (a), station 3 (b), station 4 (c), station 5 (d). Stream flow at each station plotted over a 22-month period. Compare the differences in flow for 1999 (dry year) and 2000 (wet year), particularly in stations 4 and 5. Seasonal changes are also apparent.

and 5 from December 1998 to November 2000 (excluding storm flows). Generally, flow in the stream is most constant in the section between stations 2 and 3. At stations 4 and 5, the stream undergoes greater seasonal and interannual variability. Interannual variations dominate this system; there is more change in average flow between wet and dry years than between winter and summer baseflows. In the upstream portion of the wetland, the “floodplain” serves more as a site for groundwater exfiltration than stream flooding. Fig. 4 is a plan-view topographic map of the upper portion of the riparian zone, showing field boundaries, stream channel, upwelling zones, sampling stations (1, 2, and 3), and piezometers. Only the major secondary channel system is depicted in this figure. Permanently saturated areas on the floodplain comprise about 25% of total surface area in the upper half of the riparian zone.

### Discharge per Stream Length

The amount of stream flow added between each pair of stations was normalized by intervening stream lengths to obtain flow added per unit length along each stream segment (Fig. 5). The section between stations 2 and 3 received the greatest input, and is the section that contains the largest area of permanent surface saturation and many subchannels. This section is also where the stream had its highest nitrate-N concentrations (and, at baseflow, the highest nitrate-N fluxes). The exception is during high flow periods when runoff occurs; at these times, greater flow is added between other station pairs.

Much of the increased flow between stations 2 and 3 comes from discrete sources that are visible, and often measurable. These sources are upwelling zones (concentrated areas of groundwater discharge to the surface) and associated secondary channels. There are several runnels and subchannels on the floodplain that carry upwelling groundwater to the stream. In some cases, the emergent groundwater discharges to the stream via a set of macropores along the stream channel side. Measurements taken directly from one of these, using a cup and stopwatch, indicate that approximately 10% of total flow added between stream sampling stations 2 and 3 came from a single macropore. This percentage remains fairly consistent during seasonal and other variations in baseflow conditions.

### Secondary Channels and Upwelling Zones

Where groundwater upwellings are particularly strong and concentrated, secondary channels have formed in response to the constant efflux of groundwater. The residence time for this water within the soil is thus shorter than if it were discharged more uniformly beneath the stream channel. The area between stations 2 and 3 is where the greatest number of active upwelling sites and secondary channels are found. The source of much of the stream flow here is a single large subchannel system (see Fig. 3). Periodic discharge and N-flux measurements indicate that this secondary channel system supplies an average of one third of the total flow increase between stations 2 and 3, and contributes an average of 35 to 40% to total N load exported by the stream at station 5. The total discharge (Q) carried by the secondary channel system varies less than that of the stream channel, both seasonally and in response to precipitation conditions. Even after a long drought period, flow in the secondary channel continued, while the stream channel itself was dry everywhere except near station 3.

Much of the flow generated between stations 2 and 3 was contributed by very small areas of the floodplain. While the secondary channel system supplied much of this additional flow, in actuality flow generation was even more focused than it first appeared. Most of the flow carried by the secondary channel originated in a small area of especially intense groundwater upwelling. This entire area of focused upwelling is only about 4.8 $m^2$, representing about 0.006% of the riparian area, or 0.001% of the entire catchment. The contribution of this discharge to total stream flow is around 3.5%, so obviously groundwater delivery is not uniform throughout the riparian zone.

### Preferential Flow and Groundwater Delivery

Preferential flow appears to play an extremely important role in this system, especially in the upper (headward) portion of the catchment. Although the riparian soils are much less hydraulically conductive than the underlying aquifer, there are well-defined higher-conductivity layers within the upper 2 m (the soil zone) that help transmit groundwater through the wetland sediments. Most notable are semicontinuous sand layers within the soil (at depths of approximately 90 and 130 cm below the surface) that have hydraulic conductivity values at least an order of magnitude greater than the surrounding material. Macropores may interact with subsurface flow paths and provide conduits through which groundwater can reach the surface rapidly. Preferential groundwater flow, as demonstrated at this site, can play an important role in streamflow generation, and may also account for large differences between predicted and observed degrees of denitrification.

Examination of subsurface flow paths indicates that preferential flow is a significant mechanism for groundwater (and nitrogen) delivery from the field to the wetland. The upstream part of the riparian floodplain receives a continuous and focused influx of groundwater from the adjacent agricultural field. Ground penetrating radar (GPR) data recorded in the agricultural field indicate the presence of a restricting clay layer in the subsurface (see Walthall et al.[12]). This low-permeability layer is oriented in such a way that much of the groundwater beneath a large part of the upland is directed into a narrow section of the neighboring riparian wetland. Likely pathways for preferential groundwater flow in the subsurface, based on combined GPR and surface topography data (see Walthall et al.[12]), have been mapped for the field (see Figs. 2 and 3). These flow paths appear to direct the groundwater toward the part of the riparian system where the highest surface water nitrate concentrations are found.

### Comparison with Other Portions of Riparian Zone

The downstream portion of the riparian zone differs markedly from the upper area in physical appearance, percent of total stream flow supplied, and geochemistry. Downstream, there are few upwelling zones (see Fig. 2) and active subchannels, and the stream is more incised.

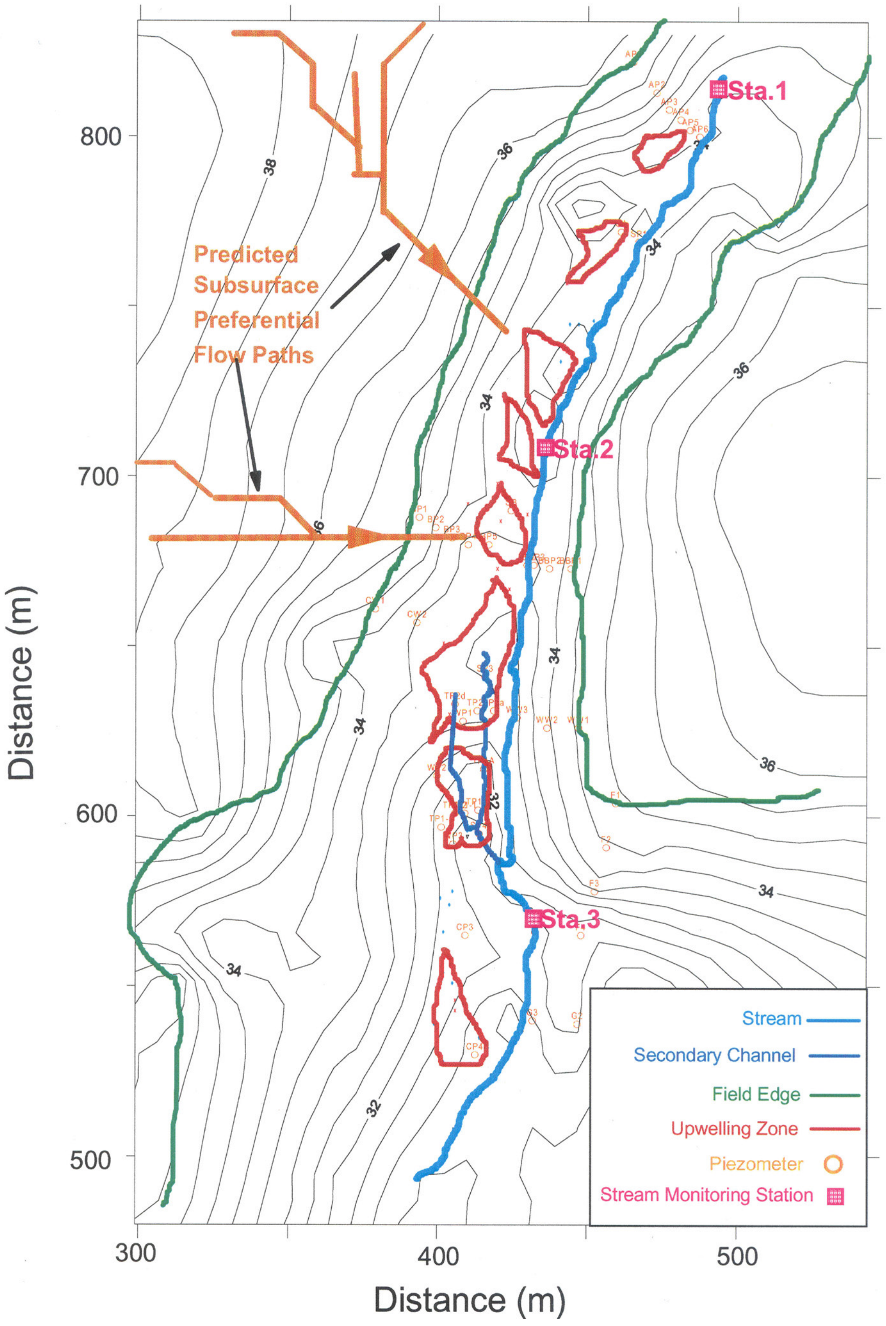

**FIGURE 4.** Close-up plan view of upper portion of study site. This figure shows upwelling zones, piezometers, and major secondary channel system. Stream channel, field boundaries, and sampling stations also shown. Upwelling zones (areas of permanent saturation) dominate the floodplain in this area. Preferential subsurface flowpaths from field to forest lead into zone of maximum exfiltration on floodplain. Nested piezometers are depicted as single wells. Coordinates are in meters from origin (see Fig. 2).

Although the groundwater coming from the field contains agricultural contamination similar to that found upstream, little or none of the nitrate here enters the stream channel during low baseflow conditions. Under higher flow conditions, though, the downstream portion of the riparian zone becomes a net source of stream N loading. In the most downstream portion of the site, the high nitrate-N concentrations (15 to 20 mg/l) detected in the near-field piezometers disappear rapidly as the groundwater progresses through the riparian zone. Piezometers near the stream channel contained very little nitrate-N, except during high-flow regimes. In addition, excess dinitrogen (particularly in relation to argon levels) was detected in these wells, evidence that denitrification was probably taking place here[13]. There were significant differences in soil between this portion of the wetland and the area further upstream. Although there is still extensive visible macroporosity, this area lacks the extensive continuous subsurface sand layers found upstream. Soil here has a higher bulk density and a greater clay component.

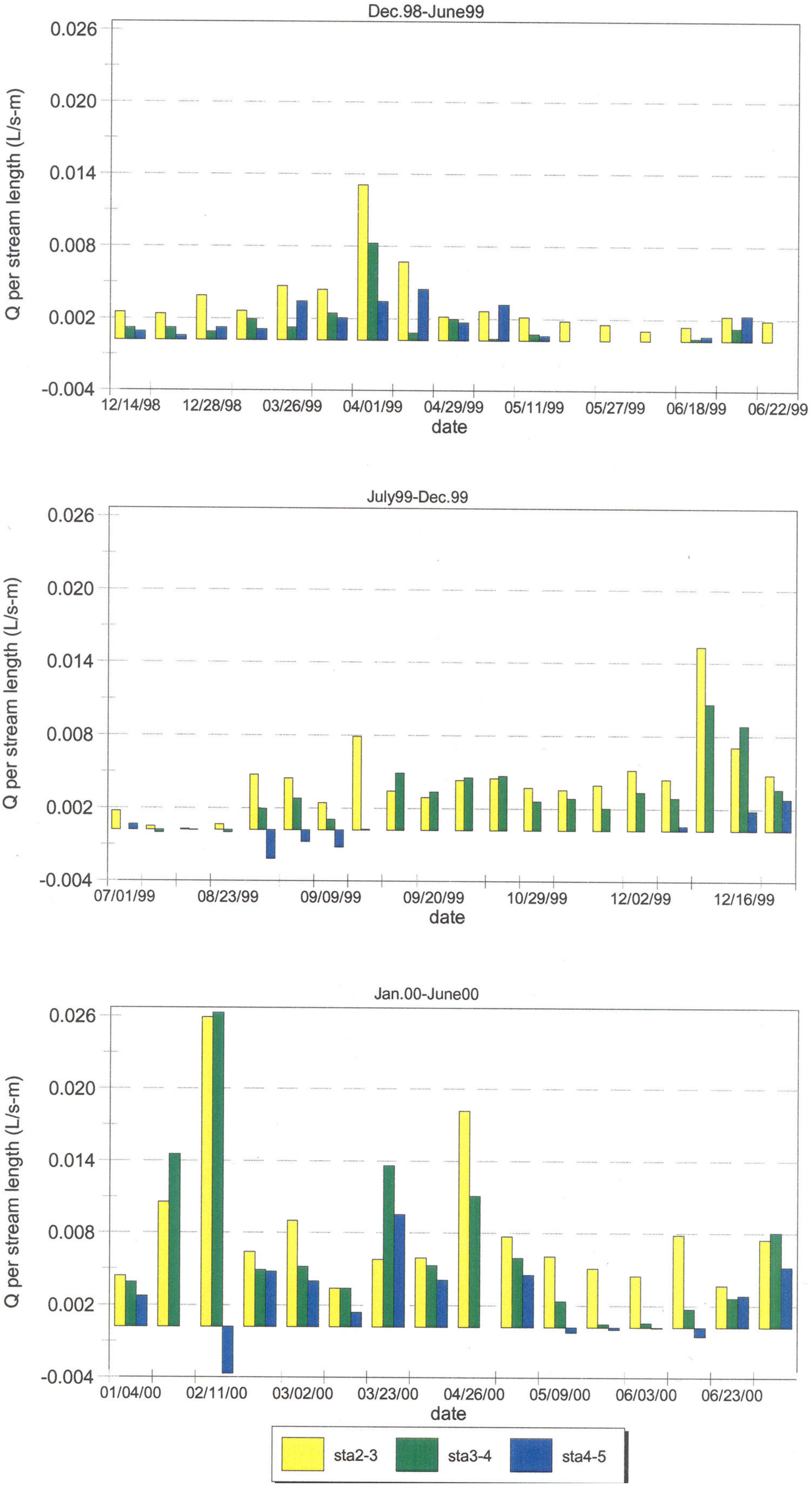

**FIGURE 5.** Discharge added per unit stream length. This figure shows stream flow added between stations per unit length over an 18-month period. By far the greatest amount of water added to the stream per unit length is between stations 2 and 3. The exception is during high streamflow, when runoff occurs. The amount of flow added is also most constant between stations 2 and 3.

### Temporal Variations in Stream Nitrate-N Concentrations

There are many variations in nitrate-N behavior in this stream system, the most prominent being interannual variations (especially between wet and dry years), seasonal variations, and diurnal changes (particularly during the growing season). It appears, however, that interannual variations are much greater than either diurnal or seasonal variations, as has been seen in other studies[10]. This study lasted more than 2 years (including three summers); in that time there have been two dry summers and one wet summer. Fig. 6 shows nitrate-N concentrations at each station for all flows from December 1998 to November 2000. Stream nitrate-N concentrations were higher in the wet year (2000) than in the dry years. The difference in nitrate-N flux was even greater (see Fig. 7). Most studies evaluating the effects of wet vs. dry years on nitrate-N have concluded that denitrification increases during wet years[9,14,15], so stream nitrate-N levels theoretically should be lower. This assumption is based in part on widely varying water table levels in the wetland. At this study site, however, water table levels do not change much within the floodplain, while they do vary substantially in the upland and hillslope. Water table levels in much of the floodplain dropped only a few centimeters in the driest year, still remaining within the upper, most biologically active zone of riparian soil. During low flows associated with dry years, contaminated groundwater moves up through the soil profile at a slower rate, so there is more contact time with the potentially denitrifying sediments. In addition, matrix flow may represent a greater proportion of subsurface and emergent flow. Flow in the secondary channels is also lower (and slower), so not only is there more contact time, but a larger percentage of the total water carried comes in contact with highly active surface material. Essentially, the surface area to volume ratio increases. The same applies to the stream; for a smaller volume of water passing through the stream channel, a larger proportion comes in contact with the channel bed. Additionally, there is no great decrease in water that seeps through the upper (highly active) soil layers because many of these areas remain saturated even during droughts, so this near-surface seepage water represents a larger percentage of total water delivered to the stream. There is also significant loss of water along the lower part of the stream in dry summers, so flux is greatly diminished. During the wet summer there was little or no net loss of flow downstream; in the severe drought year flow ceased altogether in much of the stream channel, so nitrate-N flux out of the system became zero. There are also large seasonal variations in stream nitrate-N patterns. Typically, nitrate-N concentrations are higher on average for a given year during winter (see Fig. 6). This is consistent with other studies[16,17].

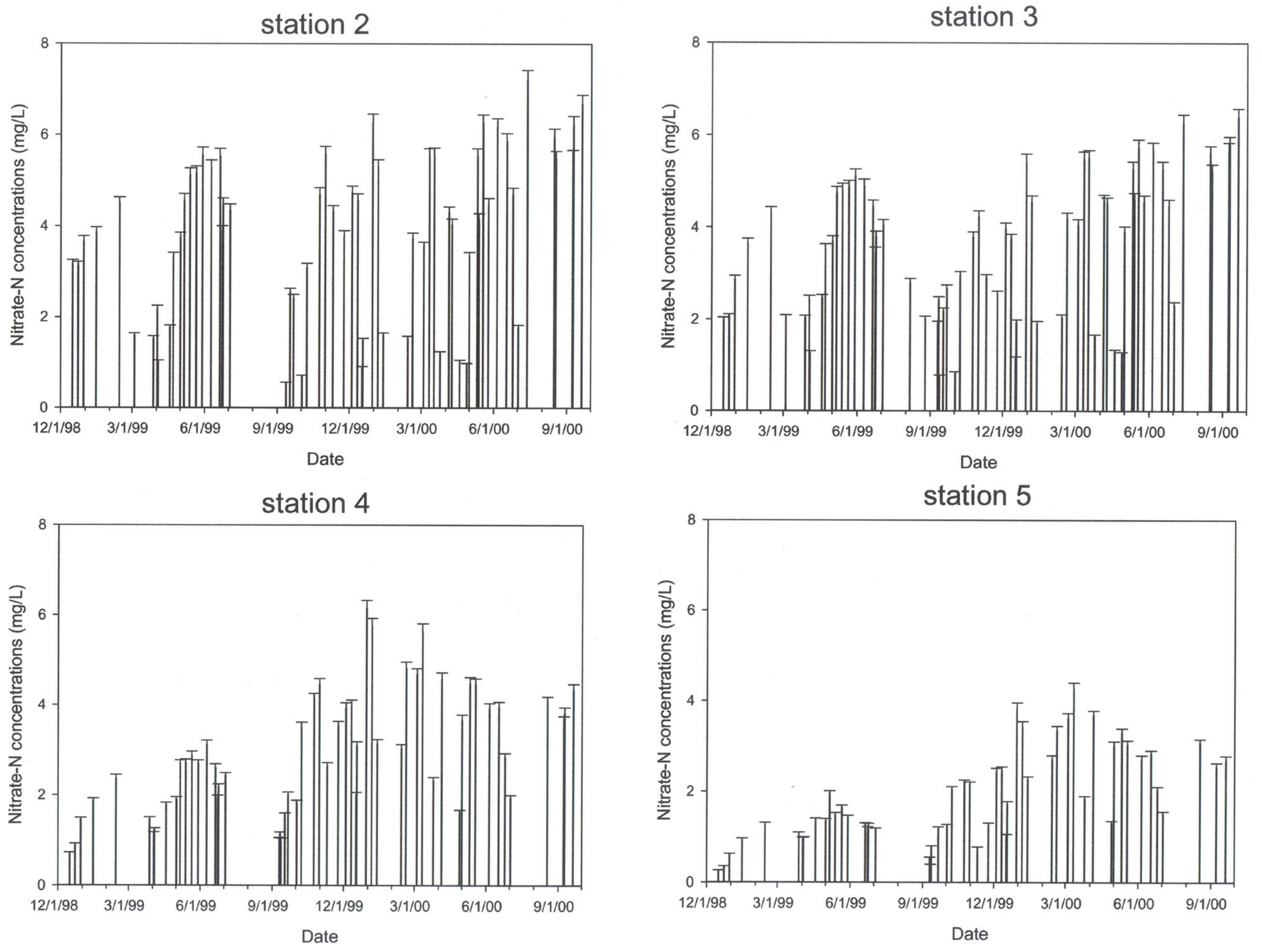

**FIGURE 6.** Stream nitrate concentrations. Nitrate-N concentrations in stream water are plotted for each station (2–5) from December 1998 to November 2000. Values are for all measurable flows (very low baseflow to near-bankfull stage). Nitrate levels in the stream were consistently higher for the wet year (2000) than for the dry year, especially in the downstream sections (stations 4 and 5).

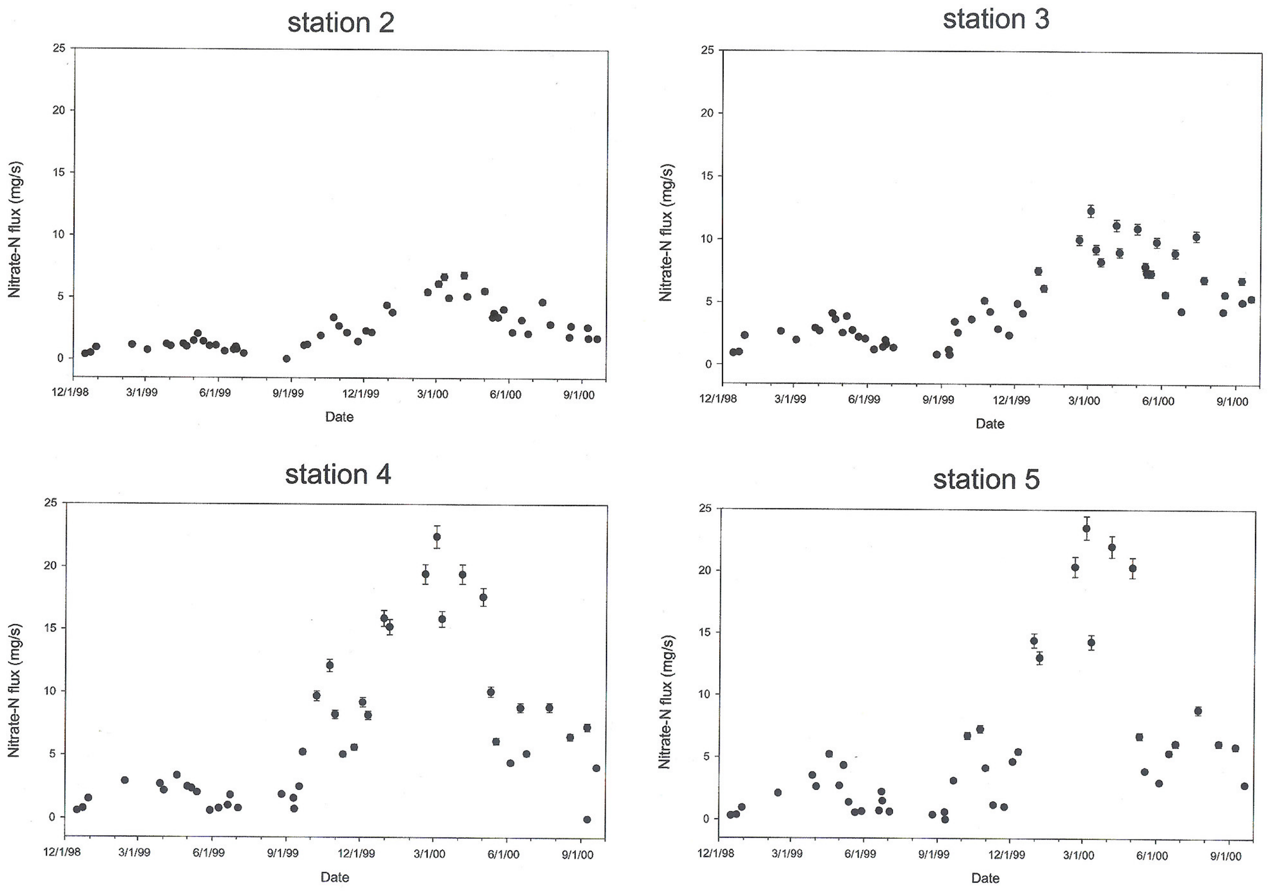

**FIGURE 7.** Stream nitrate fluxes: baseflows. Nitrate fluxes for stream baseflows at each station (2–5), from December 1998 to October 2000. Seasonal and interannual variations are discernable, especially at stations 4 and 5. Note the large differences in average fluxes for the wet year (2000) vs. the dry year (1999). Maximum nitrate flux out of the system (station 5) was five times greater in 2000 than in 1999.

### Nitrate-N Flux: Temporal and Spatial Variations

Ultimately, it is nitrate-N flux, or the quantity of nitrate-N export per time, that is of greatest importance in terms of larger environmental impact. The nitrate-N flux from each station within the stream is plotted in Fig. 7 for a period from December 1998 to October 2000. There is a notable seasonal effect, but this is vastly overwhelmed by interannual differences in rainfall. The station showing the highest flux of nitrate-N in this system varies according to total discharge. In general, for high flows, station 5 exhibited the greatest nitrate-N fluxes, while station 3 showed the highest fluxes when streamflow was very low, with station 4 highest during intermediate flows. Of course, the flux of most concern is that which leaves the system — namely, the flux of nitrate-N out of station 5. The nitrate-N load leaving the system from station 5 is by far greatest during the winter months, especially for a wet year (2000). The largest fluxes were from station 5 during February–April 2000, with maximum exported nitrate-N loads of nearly 25 mg/s. In contrast, the same period in the previous (very dry) year yielded maximum nitrate-N fluxes just over 5 mg/s. Clearly there is great variability in nitrate-N flux, largely the result of variations in rainfall amounts and distribution. For much higher stream flows, the potential for nitrate-N export from the system is far greater.

### CONCLUSIONS

The fate of nitrate-N in small groundwater-fed first-order streams is still a largely unresolved question. According to the literature, wide riparian zones associated with unincised first-order streams in low-relief areas should provide nearly ideal conditions for nitrate-N removal[1,2,3,15]. Nevertheless, in this study it is precisely near the “stream head,” where the stream channel is least incised, that we find the highest nitrate-N levels in the stream water. In this case, factors other than those typically cited must be responsible for controlling the amount of nitrate-N that reaches the stream. Our results indicate that local subsurface hydrology, and most particularly a critical amount of groundwater delivery to a given area (aided by preferential flow of many types), greatly affects the denitrification capacity of that section of riparian buffer, and consequently influences stream nitrate-N flux.

Stream flow generation at this site is clearly heterogenous. The enhanced stream flow generated between stations 2 and 3 is inconsistent with a model of homogenous flow through the riparian zone and requires a consideration of more complex processes involving preferential flow. Groundwater exfiltration to the surface is so patchy and localized at this study site that small, well-defined areas supply a disproportionate amount to total streamflow. These regions act as preferential sources for surface water.

There is a large disparity in nitrate-N delivery to different parts of the stream, which is related to groundwater delivery patterns. Sections of the riparian ecosystem where much of the groundwater emerges onto the floodplain exhibit elevated surface water nitrate-N concentrations. Areas where most of the groundwater appears to be discharged directly under the stream channel show lower nitrate-N concentrations. Therefore, at least in this setting, there is a direct correlation between groundwater delivery patterns and stream nitrate-N loads. The large differences in N observed in different stream sections appear to reflect the mechanisms (and rates) by which groundwater is fed into the stream.

| | | |
|---|---|---|
| Received: | July | 27, 2001 |
| Revised: | November | 9, 2001 |
| Accepted: | November | 9, 2001 |
| Published: | November | 21, 2001 |
